# Case Report: A case of severe pulmonary hypertension combined with FBN1 mutation associated geleophysic dysplasia

**DOI:** 10.3389/fped.2025.1642390

**Published:** 2025-07-16

**Authors:** Ze-yang Chen, Yuan Cao, Jie Yang, Xue-hua He, Li-ping Liu, Yong-hua Yuan

**Affiliations:** ^1^ School of Medicine, Qingdao University, Qingdao, China; ^2^ Department of Pediatric Cardiology, Hunan Provincial People's Hospital, The First Affiliated Hospital of Hunan Normal University, Changsha, China

**Keywords:** FBN1, geleophysic dysplasia, mitral valve lesion, pulmonary hypertension, timing of surgery 1

## Abstract

**Background:**

FBN1 gene mutation-associated geleophysic dysplasia (GD) leads to the formation of complex and refractory pulmonary hypertension (PH) through a multifactorial combination of precapillary factors, postcapillary factors, and respiratory pathology. However, clinical experience regarding the diagnosis and management of these patients remains limited.

**Case report:**

The patient was admitted to the hospital with severe PH symptom. He exhibited typical facial features, severe disproportionate short stature, and was diagnosed with GD following the identification of a heterozygous mutation in exon 42 of the FBN1 gene via whole-exome sequencing. Pulmonary artery pressure was reduced after admission and treatment with treprostinil, but mitral stenosis progressively worsened. The patient was then treated with mitral valvuloplasty + atrial septal windowing at an outside hospital, the procedure was successful, but the patient could not be weaned from ECMO after the procedure.

**Conclusion:**

This case expands our understanding of therapeutic strategies for PH associated with FBN1 mutation–related GD. Treprostinil may be effective in the treatment of these patients. Given the risk of progressive pulmonary disease, early surgical intervention for mitral valve pathology may be crucial for improving prognosis.

## 1 Introduction

Mutations in the FBN1 gene that cause skeletal dysplasias include acromicric dysplasia (AD), geleophysic dysplasia (GD), and Weill-Marchesani syndrome (WMS) (1). All three diseases are inherited in an autosomal dominant manner and are commonly characterized by short stature (< −3 SD), disproportionately short limbs, progressive joint limitation, skeletal abnormalities, and normal intelligence (2). Of these, patients with GD are distinguished from those with AD by the presence of a characteristic happy face with full cheeks, progressive thickening of the heart valves, severe and recurrent respiratory problems, hepatomegaly, and tiptoe gait (3). In addition, radiological features including short metacarpals, medial notch of the second metacarpal, lateral notch of the fifth metacarpal, and medial notch of the proximal femoral epiphysis are also present in patients with AD (4). In contrast, WMS can be distinguished from GD by its most striking feature, ocular abnormalities (5).

The most striking feature of GD is the progressive thickening of the heart valves, primarily affecting the mitral valve, followed by the aortic and pulmonary valves. Cardiac and pulmonary pathologies are usually the main cause of poor prognosis in GD (5). A previous retrospective study observed that patients with GD may present with chronic or acute pulmonary hypertension (PH), which may be associated with mitral valve disease, interstitial lung disease or upper airway pathology (6). The pathogenesis of PH in patients with GD should be analyzed from a multifactorial perspective.

This is a case report of a child presenting with severe PH as a prominent manifestation, which commenced in early childhood. The patient exhibited a combination of GD, progressive mitral stenosis, and a *de novo* mutation in the FBN1 gene, constituting a rare and complex case. Only a few cases of GD complicated by PH have been reported, and the management of these patients remains challenging due to limited experience. This case report summarizes the diagnostic and therapeutic experience of GD complicated by PH, which is of great significance for the diagnosis and management of patients with this condition.

## 2 Case present

**Figure 1:**
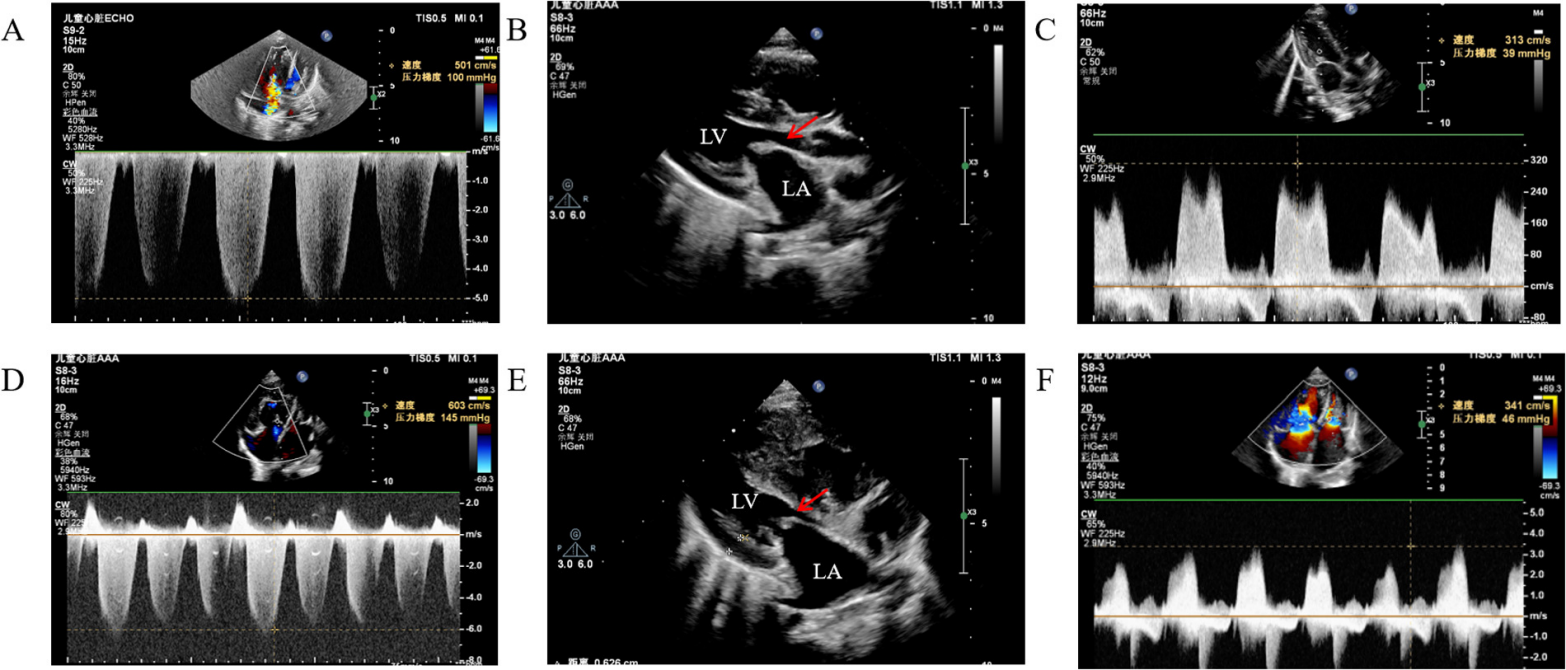
Cardiac ultrasound showed pulmonary artery pressure and progression of mitral stenosis. **(A)** pulmonary artery pressure 100 mmHg, **(B)** anterior mitral valve 3 mm, posterior valve 2.6 mm, orifice area 0.24 cm^2^, **(C)** mitral flow velocity 3.13 m/s, **(D)** pulmonary artery pressure 145 mmHg, **(E)** anterior mitral valve 3.7 mm, posterior valve 3.2 mm, orifice area 0.21 cm^2^, **(F)** mitral flow velocity 3.41 m/s. **(A-C)** results of October 2023 inspection, **(D-F)** results of December 2024 inspection.

The patient was a 2-year-3-month-old Chinese boy who presented to Hunan Provincial People's Hospital in October 2023 with cough and shortness of breath with excessive sweating and fatigue. At 7 months of age, the patient started to show excessive sweating and weakness. At 8 months of age, he was reluctant to move, usually had excessive sweating and fatigue, and snored at night and breathed with his mouth open. The patient had distinctive facial features (small nose, anteriorly tilted nostrils, wide and sunken nasal bridge, thin upper lip), puffy eyelids, hard swelling of both lower limbs, jugular vein rages, hepatomegaly (4.5 cm below the costal margin), and no splenomegaly. There was no mental retardation or stiffness of the joints of the hands. The patient presented with disproportionate short stature (78 cm, <−3 SD), low weight (9 kg, <−3 SD), short limbs, short arms and legs, and a ratio of the upper segment to the lower segment = 1.72 (+1.00 SDS). As shown in Figure 1, cardiac ultrasound results showed that the patient had severe PH with multiple valvular thickenings and enlarged right ventricle. Considering the presence of PH symptoms, the patient was given sildenafil 10 mg Q8h and bosentan 16 mg Q12h for 2 months, with no decrease in pulmonary artery pressure. Pulmonary artery pressure decreased after treatment with additional treprostinil.

**Figure 2:**
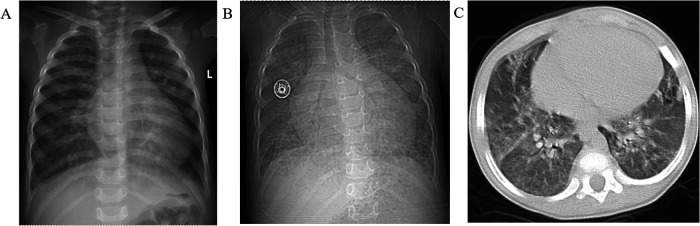
Chest radiographs showed progressive enlargement of the heart. **(A)** October 2023 results showed an enlarged heart, cardiothoracic ratio: 0.63, **(B)** December 2024 results showed further enlargement of the heart, cardiothoracic ratio: 0.69, **(C)** patient develops pulmonary edema.

Cardiac catheterization after treprostinil treatment showed pulmonary artery pressure of 100/44(62) mmHg, pulmonary capillary wedge pressure (PCWP):23/20(21) mmHg, and pulmonary small artery resistance index of 23.15 Woods. Left ventriculography showed a small left heart. Small pulmonary artery angiography showed dendritic changes in the small pulmonary arteries. As shown in Figure 2, chest radiographs showed progressive enlargement of the heart. Pulmonary artery CTA results showed no pulmonary artery stenosis or embolization, and no congenital heart disease such as VSD or pulmonary vein drainage. Laboratory test results showed that the patient's complete blood count, blood and urine tandem mass spectrometry test, thyroid function test, systemic lupus erythematosus test, and human immunodeficiency virus test were all within normal limits or negative.

**Figure 3:**
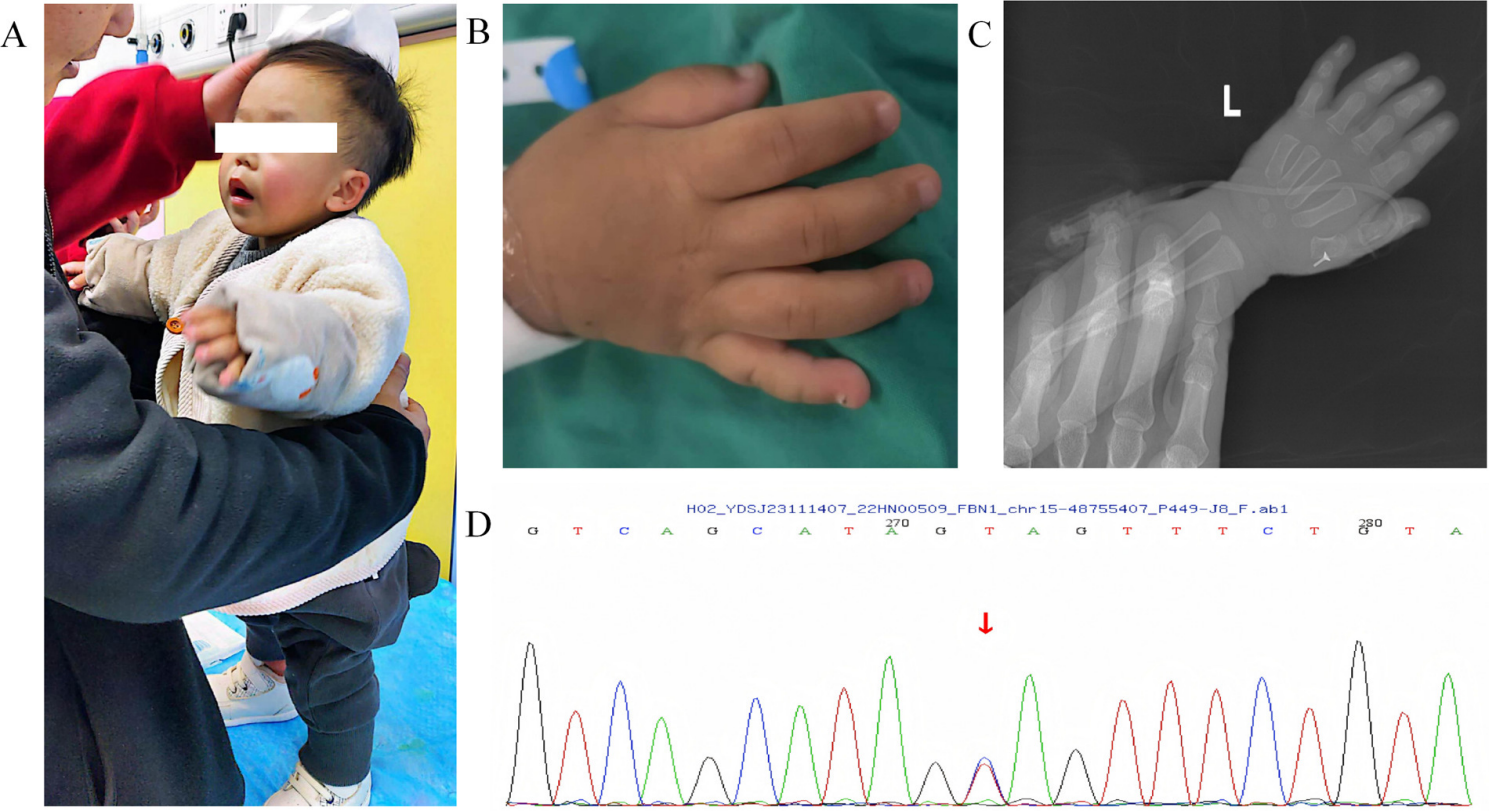
The patient had GD-related symptoms and genetic mutations. **(A-B)** the patient present with disproportionate dwarfism, short limbs, short arms and legs, **(C)** Skeletal x-ray showed delayed bone age and epiphyseal dysplasia, **(D)** whole-exome sequencing revealed *de novo* heterozygous mutations in the patient's FBN1 gene.

The patient's cardiac ultrasound findings suggested severe PH. As shown in Figure 3, his hemodynamic features were both precapillary and postcapillary PH, the etiology of her condition could not be explained by imaging and laboratory findings. In addition, the patient had typical facial features, short stature and deformities. Bone age testing revealed that the patient's bone age was 1 year or more behind his age. Lysosomal Storage Diseases associated enzyme activities tested negative, suggesting that this growth disorder may not caused by inherited metabolic disorder. To further determine the etiology, peripheral blood was collected from the patient and his parents. DNA was extracted with a DNA extraction kit, and libraries were prepared using the Agilent SureSelect All Exon kit. Library quality was assessed using Qubit 4.0 fluorometer. Paired-end exome capture was performed on an Illumina NovaSeq platform. Raw FASTQ files underwent quality control with FastQC and adapter trimming with fastp. High-quality reads were aligned to GRCh37/hg19 using BWA-MEM; PCR duplicates were marked with Picard, and base quality recalibration was done with GATK. Variants were called with GATK HaplotypeCaller and annotated via ANNOVAR and VEP against dbSNP, gnomAD, and ClinVar. Candidate variants were filtered for MAF <1% and deleterious predictions (SIFT, PolyPhen-2, CADD). Pathogenicity of the variants was determined according to American College of Medical Genetics and Genomics (ACMG) guidelines. The results revealed a heterozygous mutation in exon 42 of the patient's FBN1 gene (NM_000138.5): c.5096A > G (p.Tyr1699Cys), but none of the patient's parents had a variant at this locus, indicating a *de novo* mutation was spontaneous. According to the ACMG guidelines, this variant was initially determined to be a pathogenic variant (PS2 + PS4 + PM2_Supporting + PM5_Strong + PP3). He was ultimately diagnosed with GD-associated severe PH based on the FBN1 mutation and his cardiovascular presentation.

The patient was the first born following *in vitro* fertilization-embryo transfer (IVF-ET), and was delivered at full term with a birth weight of 2.95 kg. The family medical history showed that his parents were in good health. The grandfather died of coronary artery disease.

**Figure 4:**
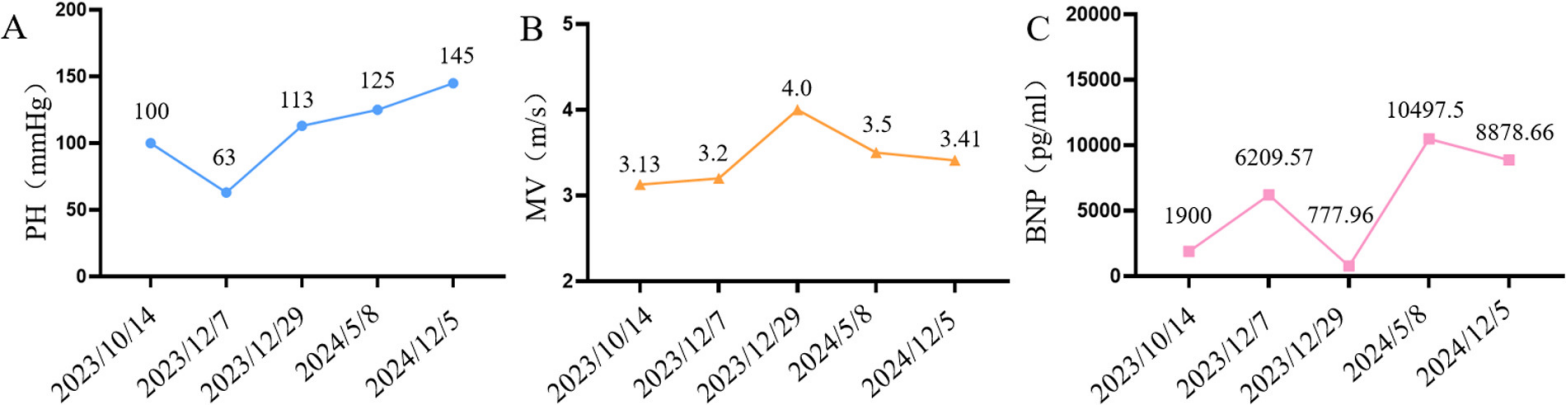
Changes in cardiovascular-related indices during patient treatment **(A)** line graph of pulmonary artery pressure changes in patients, **(B)** line graph of mitral flow velocity changes in patients, **(C)** line graph of BNP changes in patients.

As shown in Figure 4, after treatment with treprostinil (7.5 ng/kg.min), the patient's pulmonary artery pressure decreased from 100 mmHg–63 mmHg, which was effective in lowering pulmonary artery pressure. Treatment with treprostinil was continued, and after slowly increasing the dose to 15 ng/kg.min, pulmonary artery pressure rebounded to the pre-dose level and mitral flow velocity (MV) increased to 4.0 m/s, so treprostinil was discontinued. 6 months after stopping treprostinil, pulmonary artery pressure continued increase to 125 mmHg, BNP 10,497.5 (pg/ml), and the patient's shortness of breath and fatigue worsened. One year after stopping treprostinil, the pulmonary artery pressure continued increase to 145 mmHg, and the mitral valve continued to thicken, with the anterior valve thickening from 3 mm–3.7 mm, the posterior valve thickening from 2 mm–2.6 mm, and the orifice area shrinking from 0.24 cm^2^–0.21 cm^2^. Due to the severity of the mitral valve lesions, mitral valvuloplasty + atrial septal windowing was performed in an outside hospital. Although the procedure was technically successful, the patient could not be weaned from ECMO postoperatively due to severe pulmonary damage and died after the family elected to withdraw care.

## 3 Discussion

Previous studies found that GD patients carrying the FBN1 cysteine variant or the ADAMTSL2 variant have a worse prognosis and a higher likelihood of life-threatening complications, especially respiratory and heart valve diseases (4, 7). The GD patient described in this report exhibited significant progressive thickening and stenosis of the mitral valve, which may have contributed to elevated pressures in both the pulmonary veins and arteries. Moreover, cardiac catheterization showed that his pulmonary artery pressure was 100/44 mmHg, with a mean pressure of 62 mmHg, suggestive of severe PH. PCWP of 23/20 mmHg with a mean PCWP of 21 mmHg was much higher than the upper limit of normal, indicating a significant elevation of left atrial pressure overload, which may be due to mitral valve stenosis and thickening. The small pulmonary artery resistance index of 23.15 Woods indicates that the resistance of the small arteries at the end of the pulmonary circulation is extremely high, and that remodeling lesions of the small pulmonary arteries themselves (hypertrophy, fibrosis, endothelial insufficiency, etc.) are crucial in the development of PH. Therefore, the patient was diagnosed with mixed severe PH with both postcapillary and precapillary pathology (8).

In addition, this GD patient had a pathogenic mutation c.5096A > G (p.Tyr1699Cys) in exon 42 of the FBN1 gene. Most GD-associated FBN1 mutations are located within the TGF-β-binding protein-like domain 5 (TB5), which is critical for TGF-β regulation. TGF-β is a potent regulator of extracellular matrix synthesis, playing a key role in vascular remodeling and cardiac pathology in PH (9). FBN1 encodes the Fibrillin-1 protein which is a major structural component of microfibrils and constitutes the periphery of the elastic fibers. Mutations in the FBN1 gene may lead to a loss of elastic fibre integrity which weakens the vessel wall (10). Disruption of BMPR2, which interacts with FBN1 and TGF-β, impairs TGF-β and BMP4-mediated elastic fiber assembly, which has pathophysiological implications in PH. In a Bmpr2/1a heterozygous mouse model, reduction of pulmonary artery Fibrillin-1 leads to increased sensitivity of elastic fibers to degradation as well as exacerbation of PH symptoms (11). Patients with Myhre syndrome—another monogenic disorder—harbor SMAD4 mutations and exhibit skin lesions, vascular calcification, and cardiovascular abnormalities (12). The excessive deposition of vascular smooth muscle cells and cardiac extracellular matrix driven by TGF-β/SMAD hyperactivation parallels the destruction of elastic fibers caused by FBN1 mutations (13, 14). These observations suggest that abnormalities in connective-tissue components (elastic fibers and collagen), together with dysregulation of the TGF-β pathway and its downstream signaling, may be central drivers of idiopathic or hereditary cardiovascular pathologies in children.

Pathophysiologic similarities exist between GD and lysosomal storage disease. The impaired organization of the extracellular matrix observed in GD may be associated with broader cellular dysfunction involving the lysosomal pathway, which may affect the processing or degradation of glycoproteins and lead to their accumulation. This widespread cellular defect may directly contribute to the thickening and stiffening of tissues in a variety of organs, including cardiac valves and airways, thereby contributing to the pathogenesis of PH through mechanical factors (7, 15).

In addition, microfibrils, composed of Fibrillin-1, play a critical role in maintaining the structural integrity and elasticity of airway and lung tissues by providing a structural scaffold that helps maintain the mechanical stability and elasticity of the trachea, bronchi and alveolar septa (16). A previous case report showed that patients with GD can present with recurrent respiratory infections accompanied by bronchopneumonia manifestations, and imaging results suggesting severe airway stenosis (17). Although no significant airway stenosis was found in our reported GD patient, chest radiographs showed that he had developed significant pulmonary edema.

In this GD patient, the coexistence of mitral valve disease and systemic connective tissue abnormalities resulting from FBN1 mutations may exert synergistic effects that culminate in severe and refractory PH. Mitral valve stenosis elevates left atrial pressure, leading to passive pulmonary venous hypertension, whereas airway obstruction causes chronic hypoxemia and subsequent reactive pulmonary vasoconstriction. These mechanisms further exacerbate intrinsic pulmonary vascular remodeling driven by aberrant TGF-β signaling and defective elastic fibers. The complex interplay of these factors results in an increased pulmonary arterial pressure burden and a poorer prognosis, posing significant challenges for comprehensive diagnostic and therapeutic decision-making.

The patient initially received sildenafil (a phosphodiesterase-5 inhibitor) and bosentan (an endothelin receptor antagonist), but pulmonary arterial pressure did not improve. Treprostinil was subsequently added, which led to a transient reduction in pulmonary pressures. However, these pressures ultimately rebounded to pretreatment levels, accompanied by worsening mitral stenosis and cardiac enlargement. Short-term application of treprostinil significantly reduced pulmonary artery pressure, but its prolonged use increased left atrial regurgitation and exacerbated the relative degree of mitral stenosis, further contributing to increased postcapillary pressure (18). The treatment of this patient with treprostinil was overall effective, but the progressive worsening of mitral stenosis and cardiac enlargement limited its use. Moreover, after discontinuation of treprostinil, the patient experienced further increase in pulmonary artery pressure and aggravation of mitral stenosis, so mitral valvuloplasty was necessary to save the patient's life. The patient underwent mitral valvuloplasty and atrial septal windowing at an outside hospital. Mitral valvuloplasty was able to relieve the obstruction of left heart flow, thereby reducing pulmonary venous pressure and relieving post-capillary pulmonary hypertension (19). Atrial septal windowing was used to reduce right ventricular pressure and improve systemic hemodynamics by creating a right-to-left shunt between the atria, and was indicated in patients with ineffective pharmacologic therapy and symptoms of right heart failure (20). Although the procedure was very successful overall, the patient in this case was ultimately unable to be weaned off ECMO, suggesting that he may have had very severe lung disease.

Therefore, during the course of this case review, we repeatedly discussed strategies to improve the patient's prognosis and summarized our clinical experiences and lessons learned. First, the patient exhibited extremely elevated pulmonary artery pressures, which were significantly reduced following treprostinil therapy, demonstrating short-term efficacy. However, prolonged treatment with treprostinil can lead to aggravation of mitral stenosis, so the treatment of mitral stenosis using mitral valvuloplasty + atrial septal windowing and improvement of systemic hemodynamics was the right approach. Previous studies have shown that patients with FBN1 mutation-associated GD are typically born with normal respiratory function but gradually develop airway narrowing, restrictive pulmonary physiology, interstitial lung disease, and ultimately face an increased risk of respiratory failure as they grow. They reported 5 patients with GD associated with FBN1 mutations who had concomitant heart valve disease with PH, 4 patients were treated with valve replacement surgery. 3 patients were treated surgically between the ages of 7 months and 1 year, and 2 of them died at the ages of 5 and 20 years, respectively. Another patient was treated surgically at the age of 8 years and followed up until the age of 12 years (6). Most of the patients in this study underwent surgical treatment before the age of 1 year, when patients' respiratory function was less affected by the FBN1 mutation. Although 2 of these patients eventually died, early surgical treatment significantly prolonged their survival. In another case report, a patient with GD presented with symptoms of PH at the age of 14 and immediately underwent balloon pulmonary valvuloplasty, which significantly reduced the pulmonary gradient (21). Another GD patient had laryngotracheomalacia, subglottic stenosis, and restrictive lung disease. She underwent laryngotracheal reconstruction to improve respiratory symptoms. She was diagnosed with PH at the age of 6 and subsequently underwent aortic membrane resection and mitral valve replacement. Although the surgery was successful, she experienced a prolonged and difficult postoperative recovery and ultimately died of respiratory complications at the age of 8 (4). All of this evidence indicates that mitral valve surgery can significantly reduce pulmonary artery pressure, but postoperative respiratory complications remain a major contributor to patient mortality. Considering that respiratory lesions in GD patients tend to worsen with age, surgical intervention should be performed as soon as PH symptoms appear, while respiratory involvement is still relatively mild. For this reason, the patient reported here underwent surgery at 3 years and 3 months of age, by which time his pulmonary lesions had already progressed significantly, ultimately preventing successful weaning from ECMO despite technically successful surgery. Therefore, earlier mitral valve surgery may improve prognosis in such cases.

Furthermore, the markedly elevated pulmonary vascular resistance in this patient indicated severe vascular remodeling within the pulmonary arteries, and the right ventricular load was so great under such high resistance conditions that surgical interventions to lower left atrial pressure alone may be insufficient, requiring targeted pharmacological or surgical interventions in the small pulmonary arteries. Although the skeletal phenotypes of GD and Marfan syndrome differ, both share underlying mechanisms involving dysregulation of FBN1 and TGF-β signaling. In a mouse model of Marfan syndrome, losartan treatment significantly reduced the rate of aortic root dilation, improved the structural integrity of elastic fibers and suppressed TGF-β signaling activity (22). This provided new ideas for exploring similar targeted therapies for PH in GD. Lung or heart-lung transplantation remains the only definitive treatment for patients with end-stage PH unresponsive to pharmacological and surgical therapies. However, the complexity, donor scarcity, and long-term postoperative management challenges of pediatric lung transplantation are enormous (23).

## 4 Conclusion

The tragic outcome of this patient with FBN1 mutation–associated GD and PH underscores the complexity of the disease and the limitations of current therapeutic options. As FBN1 gene mutations lead to multiple pathologic changes including precapillary factors, postcapillary factors, and progressive lung lesions, their treatment options require comprehensive consideration. Although treprostinil and mitral valvuloplasty alleviated the symptoms of PH, the severity of pulmonary involvement may have contributed to the patient's inability to be weaned from ECMO. Therefore, early surgical intervention may be critical in improving the prognosis of PH patients combined with FBN1 mutation associated GD.

## Data Availability

The original contributions presented in the study are included in the article/Supplementary Material, further inquiries can be directed to the corresponding author.
